# Frequency, Risk Factors, and Clinical Outcomes of Late-Onset Atrial Flutter in Patients after Heart Transplantation

**DOI:** 10.3390/jcdd9100337

**Published:** 2022-10-03

**Authors:** Ann-Kathrin Rahm, Susanne Reinhardt, Matthias Helmschrott, Fabrice F. Darche, Tom Bruckner, Patrick Lugenbiel, Dierk Thomas, Philipp Ehlermann, Wiebke Sommer, Gregor Warnecke, Norbert Frey, Rasmus Rivinius

**Affiliations:** 1 Department of Cardiology, Angiology and Pneumology, Heidelberg University Hospital, 69120 Heidelberg, Germany; 2 Heidelberg Center for Heart Rhythm Disorders (HCR), Heidelberg University Hospital, 69120 Heidelberg, Germany; 3 German Center for Cardiovascular Research (DZHK), Partner Site Heidelberg/Mannheim, University of Heidelberg, 69120 Heidelberg, Germany; 4 Institute for Medical Biometry, University of Heidelberg, 69120 Heidelberg, Germany; 5 Department of Cardiac Surgery, Heidelberg University Hospital, 69120 Heidelberg, Germany

**Keywords:** atrial flutter, graft rejection, heart transplantation, immunosuppression, mortality, survival, tricuspid regurgitation

## Abstract

Aims: Atrial flutter (AFL) is a common late-onset complication after heart transplantation (HTX) and is associated with worse clinical outcomes. Methods: This study investigated the frequency, risk factors, and outcomes of late-onset post-transplant AFL. We analyzed 639 adult patients undergoing HTX at the Heidelberg Heart Center between 1989 and 2019. Patients were stratified by diagnosis and type of late-onset post-transplant AFL (>90 days after HTX). Results: A total of 55 patients (8.6%) were diagnosed with late-onset post-transplant AFL, 30 had typical AFL (54.5%) and 25 had atypical AFL (45.5%). Patients with AFL were younger at HTX (*p* = 0.028), received more biatrial anastomosis (*p* = 0.001), and presented with moderate or severe tricuspid regurgitation (56.4%). Typical AFL was associated with graft rejection (*p* = 0.016), whereas atypical AFL was associated with coronary artery disease (*p* = 0.028) and stent implantation (*p* = 0.042). Patients with atypical AFL showed a higher all-cause 1-year mortality (*p* = 0.010) along with a higher rate of graft failure after diagnosis of AFL (*p* = 0.023). Recurrence of AFL was high (83.6%). Patients with catheter ablation after AFL recurrence had a higher 1-year freedom from AFL (*p* = 0.003). Conclusions: Patients with late-onset post-transplant AFL were younger at HTX, received more biatrial anastomosis, and showed a higher rate of moderate or severe tricuspid regurgitation. Typical AFL was associated with graft rejection, whereas atypical AFL was associated with myocardial ischemia, graft failure, and mortality. Catheter ablation represents a viable option to avoid further episodes of late-onset AFL after HTX.

## 1. Introduction

Heart transplantation (HTX) has been considered the preferred treatment for patients with irreversible end-stage heart failure for more than 50 years [1,2,3,4,5]. As survival after HTX has continuously been improving, post-transplant clinical management is facing an increasing number of HTX recipients with chronic complications such as cardiac allograft vasculopathy, malignancy, renal dysfunction, diabetes mellitus, and cardiac arrhythmias [6,7,8,9,10,11,12,13,14].

Heart rhythm disorders after HTX may be especially amenable to promising therapeutic approaches to improve post-transplant survival and quality of life as they comprise a broad spectrum of cases [13,14,15,16,17,18,19,20]. Common cardiac arrhythmias after HTX include sinus tachycardia, sick sinus syndrome, atrioventricular block, right bundle branch block, atrial fibrillation, and atrial flutter [13,14,15,16,17,18,19,20]. While post-transplant atrial fibrillation often occurs in the early stage after HTX, atrial flutter (AFL) is a common atrial arrhythmia late after HTX [20]. The late onset of AFL after HTX may be the result of chronic changes caused by cardiac allograft vasculopathy and repeated graft rejection episodes [20,21,22,23,24,25]. Several authors have described an association between late-onset AFL after HTX and recurring episodes of graft rejection which can lead to myocardial damage including inflammation, edema, fibrosis and scar tissue resulting in cardiac remodeling as well as graft dysfunction [20,21,22,23,24,25]. Areas of atrial fibrosis and scar tissue provide a damaging electrophysiological milieu most conducive to AFL [20,21,22,23,24,25]. In addition, the risk of late-onset AFL is increased in HTX recipients with biatrial anastomosis as this HTX technique results in enlarged atrial cavities with distorted anatomy and two long surgical suture lines of donor and recipient atria providing a highly proarrhythmic substrate [17,20,21,22,23,24,25,26].

The traditional definition of AFL is based on 12-lead electrocardiogram (ECG) morphology and involves typical and atypical AFL [27]. Typical AFL is the most frequent cavotricuspid isthmus-dependent flutter cycling around the tricuspid anulus, with the cavotricuspid isthmus as the critical isthmus. The terms non-cavotricuspid isthmus-dependent macro-reentrant tachycardia and atypical flutter are used synonymously and describe flutter waves in the ECG that are not suggestive of typical macro-reentrant circuits [27].

Given the risk profile of HTX recipients with late-onset AFL, these patients are at high risk for post-transplant morbidity and mortality. However, data on the clinical management of patients with late-onset post-transplant AFL are limited and even less is known about the differences between typical and atypical late-onset AFL after HTX. We therefore sought to investigate the risk factors, treatment, and clinical outcomes of late-onset AFL after HTX with special focus on typical and atypical AFL.

## 2. Patients and Methods

### 2.1. Patients

We performed this study in accordance with the ethical standards of the Declaration of Helsinki. The institutional review board (IRB) of Heidelberg University gave approval (ethics approval number: S-286/2015, Version 1.2, 28 July 2020). We obtained written informed consent from patients for their inclusion in the Heidelberg HTX Registry and the clinical and scientific use of their data. The ethics approval does not require additional consent for this observational study as only routine clinical data were used [11,12,13,14,15,16,17,18,19].

Our study included all adult patients (≥18 years) who received HTX at the Heidelberg Heart Center, Heidelberg, Germany, between 1989 and 2019, except for patients who had undergone repeat HTX. We initially stratified patients by diagnosis of late-onset post-transplant AFL (>90 days after HTX). Patients with late-onset post-transplant AFL were further divided into patients with typical and atypical AFL based on 12-lead ECG findings and electrophysiological study (EPS) data in case of performed ablation [27]. The traditional definition of AFL in general according to the 12-lead ECG morphology is as follows: continuous regular electrical activity, most commonly a saw-tooth pattern in contrast to focal atrial tachycardia, with isoelectric lines in between P-waves. [27].

### 2.2. Follow-Up

Patient follow-up was performed in accordance with the Heidelberg Heart Center’s routine clinical protocol. After the initial hospital stay following HTX, patients were seen monthly as outpatients in the HTX clinic during the first six post-transplant months, then bimonthly until the end of the first year after HTX, and approximately three to four times per year thereafter (with additional visits on demand) [11,12,13,14,15,16,17,18,19].

Routine follow-up included medical history, physical examination, systolic and diastolic blood pressure measurement, blood and laboratory tests including immunosuppressive drug monitoring, resting 12-lead ECG, echocardiography, endomyocardial biopsy, annual chest X-ray as well as annual 24-h Holter monitoring. We were able to obtain complete follow-up data after HTX from all patients as no patient was lost to follow-up. In addition, we could record all causes of death within one year after diagnosis of late-onset AFL after HTX [11,12,13,14,15,16,17,18,19].

### 2.3. Post-Transplant Medications

Post-transplant medications including immunosuppressive drug therapy were administered as per the Heidelberg Heart Center’s standard of care. Perioperatively, patients received an anti-thymocyte globulin-based immunosuppression induction therapy. Cyclosporine A and azathioprine were applied as the initial immunosuppression until 2001. Mycophenolate mofetil consequently replaced azathioprine from 2001 onward, and tacrolimus subsequently replaced cyclosporine A since 2006. Steroids were tapered incrementally during the initial post-transplant months and were discontinued six months after HTX (unless clinically needed) [11,12,13,14,15,16,17,18,19].

### 2.4. Statistical Analysis

The primary outcome of this study was 1-year mortality after diagnosis of late-onset post-transplant AFL, which was further assessed by stratification into patients with typical and atypical late-onset post-transplant AFL. Causes of death within one year after diagnosis of late-onset post-transplant AFL were categorized into the following groups: graft failure, acute rejection, infection/sepsis, malignancy, and thromboembolic event/bleeding [11,12,13,14,15,16,17,18,19].

Secondary outcomes included analysis of clinical findings, risks factors and treatment of patients with late-onset AFL after HTX. Our analysis comprised multiple univariate analyses in order to search for intergroup differences between patients with and without diagnosis of late-onset AFL after HTX as well as between patients with typical and atypical late-onset AFL after HTX. Parameters included recipient data, recipient previous open-heart surgery, recipient principal diagnosis for HTX, donor data, transplant sex mismatch, perioperative data, immunosuppressive drug therapy, and post-transplant concomitant medications [11,12,13,14,15,16,17,18,19].

Patients with late-onset AFL after HTX were further analyzed with regard to clinical presentation and findings, echocardiographic features, and acute graft rejection (diagnosed ± 7 days at the time of AFL), as well as treatment modalities. Given the long study period of more than 30 years, we performed a sensitivity analysis to test the robustness of our results and to examine a possible era effect using a subgroup of patients with tacrolimus and mycophenolate mofetil, since the immunosuppressive drug regimen was changed from 2006 onward [11,12,13,14,15,16,17,18,19].

Data were analyzed using SAS (Version 9.4, SAS Institute, Cary, NC, USA) and shown as mean ± standard deviation (SD), median with quartiles (Q), or as count (*n*) with percentage (%). For measures of association, difference of mean or hazard ratio (HR) with 95% confidence interval (CI) were applied. Depending on the variable type and question, we used Student’s *t*-test, Mann–Whitney U-test, analysis of variance (ANOVA), Kruskal–Wallis test, chi-squared test, or Fisher’s exact test, as appropriate. The Kaplan–Meier estimator was used to graphically compare 1-year survival after diagnosis of late-onset post-transplant AFL in patients with typical and atypical AFL as well as to analyze 1-year freedom from further episodes of late-onset AFL after HTX between patients with and without catheter ablation after recurrence of late-onset post-transplant AFL. A *p*-value of <0.050 was considered statistically significant [11,12,13,14,15,16,17,18,19].

## 3. Results

### 3.1. Demographics of Patients with and without Late-Onset Post-Transplant Atrial Flutter

Out of 639 included HTX recipients, 55 patients (8.6%) were diagnosed with late-onset post-transplant AFL. Of these, 30 HTX recipients (30 of 55 [54.5%]) had typical late-onset AFL after HTX, and 25 HTX recipients (25 of 55 [45.5%]) had atypical late-onset AFL after HTX. The median interval from HTX to the initial diagnosis of late-onset post-transplant AFL was 8.8 years (Q1: 4.7 years; Q3: 14.6 years) and the median interval from diagnosis of late-onset post-transplant AFL until last follow-up was 2.3 years (Q1: 1.1 years; Q3: 5.8 years). Patients with late-onset AFL after HTX had a significantly lower recipient age at HTX (48.8 ± 11.2 years versus 52.4 ± 10.2 years; *p* = 0.028) and a significantly higher percentage of biatrial anastomosis (43.6% versus 24.0%; *p* = 0.001), whereas patients without diagnosis of late-onset AFL after HTX showed a significantly higher percentage of bicaval anastomosis (76.0% versus 56.4%; *p* = 0.001). Demographics of study participants are shown in Table 1.

Analysis of demographics between patients with typical and atypical late-onset AFL after HTX showed that typical AFL was more present in male HTX recipients (86.7% versus 64.0%; *p* = 0.049), while atypical AFL was associated with a significantly higher percentage of arterial hypertension (68.0% versus 40.0%; *p* = 0.038). Demographics stratified by typical and atypical late-onset AFL after HTX are presented in Table 2.

### 3.2. Medications after Heart Transplantation

In terms of the immunosuppressive drug therapy, we found no statistically significant differences between patients with and without diagnosis of late-onset AFL after HTX concerning the use of cyclosporine A, tacrolimus, azathioprine, or mycophenolate mofetil (all *p* ≥ 0.050). We also observed no statistically significant differences between patients with and without diagnosis of late-onset AFL after HTX regarding the administration of acetylsalicylic acid, beta blockers, ivabradine, calcium channel blockers, angiotensin-converting-enzyme inhibitors/angiotensin II receptor blockers, or statins (all *p* ≥ 0.050). Medications of study participants are given in Table 3.

Likewise, there were no statistically significant differences between patients with typical and atypical late-onset AFL after HTX concerning immunosuppressive drugs or concomitant medications (all *p* ≥ 0.050). Medications stratified by typical and atypical late-onset AFL after HTX are shown in Table 4.

### 3.3. Clinical Presentation and Findings of Patients with Late-Onset Post-Transplant Atrial Flutter

The majority of patients with late-onset post-transplant AFL were symptomatic (46 of 55 [83.6%]) but patients with typical AFL were more symptomatic than patients with atypical AFL (93.3% versus 72.0%; *p* = 0.033). Patients with typical AFL had a higher percentage of palpitations (86.7% versus 60.0%; *p* = 0.024) and AFL with 2:1 atrioventricular conduction (66.7% versus 36.0%; *p* = 0.023), while patients with atypical AFL showed a higher percentage of chest pain (28.0% versus 6.7%; *p* = 0.033) and peripheral edema (56.0% versus 26.7%; *p* = 0.027). Late-onset post-transplant AFL was incidentally detected in 9 of 55 patients (16.4%), either on routine resting 12-lead ECG (7 of 55 [12.7%]) or on routine 24-h Holter monitoring (2 of 55 [3.6%]). Especially on routine resting 12-lead ECG, asymptomatic atypical AFL was significantly more often found than asymptomatic typical AFL (24.0% versus 3.3%; *p* = 0.022). Regarding clinical findings, patients with atypical AFL suffered from a significantly higher percentage of coronary artery disease (52.0% versus 23.3%; *p* = 0.028), acute myocardial ischemia with requirement for coronary stent implantation (32.0% versus 10.0%; *p* = 0.042), acute infection (32.0% versus 10.0%; *p* = 0.042), and chronic hemodialysis (32.0% versus 10.0%; *p* = 0.042), whereas patients with typical AFL had a significantly higher percentage of acute graft rejection (46.7% versus 16.0%; *p* = 0.016). Clinical presentation and findings of patients with late-onset AFL after HTX are presented in Table 5.

### 3.4. Echocardiographic Features of Patients with Late-Onset Post-Transplant Atrial Flutter

Assessment of echocardiographic features showed that HTX recipients with late-onset post-transplant AFL had a high percentage of an enlarged right atrial (81.8%) and right ventricular diameter (70.9%) along with an elevated rate of reduced right ventricular function in more than half of all patients (52.7%). Comparison between patients with typical and atypical late-onset AFL after HTX indicated a higher rate of impaired left-sided heart function in patients with atypical late-onset AFL after HTX. They had a higher rate of a reduced left ventricular function (15 of 25 [60.0%] versus 5 of 30 [16.7%]; *p* = 0.001), a higher rate of mitral regurgitation (21 of 25 [84.0%] versus 17 of 30 [56.7%]; *p* = 0.029), a larger left atrial diameter (46.0 ± 5.8 mm versus 42.2 ± 4.6 mm; *p* = 0.011), a larger left ventricular diameter (52.8 ± 5.9 mm versus 49.2 ± 4.6 mm; *p* = 0.019), and a reduced mitral annular plane systolic excursion (MAPSE; 12.1 ± 3.6 mm versus 14.5 ± 2.5 mm; *p* = 0.007).

Analysis of diastolic parameters showed that patients with atypical late-onset AFL after HTX had a higher early diastolic mitral inflow peak velocity (E) to late diastolic mitral inflow peak velocity (A) ratio (E/A; 3.1 ± 1.0 versus 2.3 ± 0.7; *p* = 0.002), a higher early diastolic mitral inflow peak velocity (E) to early diastolic mitral annular velocity (e′) ratio (E/e′; 15.1 ± 4.7 versus 7.7 ± 3.4; *p* < 0.001), a lower deceleration time (DT) of the early diastolic mitral inflow peak (E) (DT-E; 153.4 ± 27.6 ms versus 201.7 ± 26.8 ms; *p* < 0.001), a higher systolic pulmonary artery pressure (systolic PAP; 39.6 ± 10.2 mmHg versus 32.6 ± 9.9 mmHg; *p* = 0.013), a higher right atrial pressure (RAP; 12.2 ± 6.0 mmHg versus 8.2 ± 4.3 mmHg; *p* = 0.007), and a more dilated inferior vena cava (IVC; 23.6 ± 6.0 mm versus 19.7 ± 5.0 mm; *p* = 0.013). Echocardiographic features of patients with late-onset AFL after HTX are given in Table 6.

### 3.5. Treatment of Patients with Late-Onset Post-Transplant Atrial Flutter

At the initial occurrence of late-onset AFL after HTX, the majority of patients received either electrical (39 of 55 [70.9%]) or pharmacological cardioversion (9 of 55 [16.4%]) as treatment.

Patients with typical AFL received significantly more often electrical cardioversion (86.7% versus 52.0%; *p* = 0.005), while patients with atypical AFL received significantly more often pharmacological cardioversion (28.0% versus 6.7%; *p* = 0.033). No patient received catheter ablation at the initial occurrence of late-onset AFL after HTX, and spontaneous conversion of AFL only happened in a minority of patients (7 of 55 [12.7%]). Most patients with late-onset AFL after HTX had recurrence of AFL (46 of 55 [83.6%]). Preferred choice of treatment for patients with recurrence of late-onset AFL after HTX was catheter ablation which was performed in 20 patients (13 patients with typical AFL and 7 patients with atypical AFL; *p* = 0.239). By contrast, 15 patients received electrical cardioversion (12 patients with typical AFL and 3 patients with atypical AFL; *p* = 0.020) and 7 patients received pharmacological cardioversion (1 patient with typical AFL and 6 patients with atypical AFL; *p* = 0.022).

**Figure 1:**
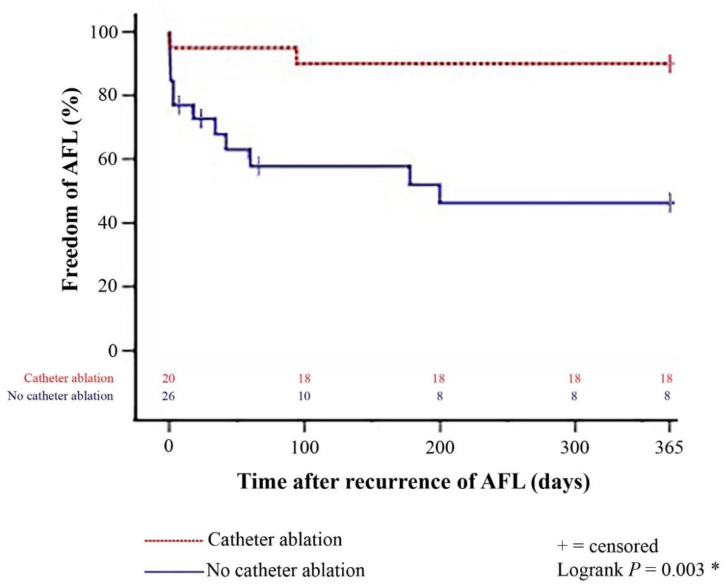
One-year freedom from further episodes of post-transplant AFL stratified by patients with and without catheter ablation after recurrence of late-onset post-transplant AFL (Kaplan–Meier estimator). Patients with catheter ablation after recurrence of late-onset post-transplant AFL had a significantly lower 1-year rate of further episodes of post-transplant AFL than patients without catheter ablation (*p* = 0.003). AFL = atrial flutter; * = statistically significant (*p* < 0.050).

Comparison between patients with and without catheter ablation after recurrence of late-onset post-transplant AFL showed a significantly lower 1-year rate of further episodes of post-transplant AFL in patients with catheter ablation (*p* = 0.003). The Kaplan–Meier estimator of 1-year freedom from further episodes of post-transplant AFL stratified by patients with and without catheter ablation after recurrence of late-onset post-transplant AFL is presented in Figure 1. Further episodes of late-onset AFL after HTX occurred in about one-third of all patients (19 of 55 [34.5%]). Of these, 7 patients received pharmacological cardioversion (1 patient with typical AFL and 6 patients with atypical AFL; *p* = 0.022), 5 patients received electrical cardioversion (4 patients with typical AFL and 1 patient with atypical AFL; *p* = 0.231), 2 patients received catheter ablation (2 patients with typical AFL and 0 patients with atypical AFL; *p* = 0.188), and 2 further patients received repeat catheter ablation (1 patient with typical AFL and 1 patient with atypical AFL; *p* = 0.895). Treatment of patients with late-onset AFL after HTX is summarized in Table 7.

### 3.6. Mortality and Causes of Death after Diagnosis of Late-Onset Post-Transplant Atrial Flutter

**Figure 2:**
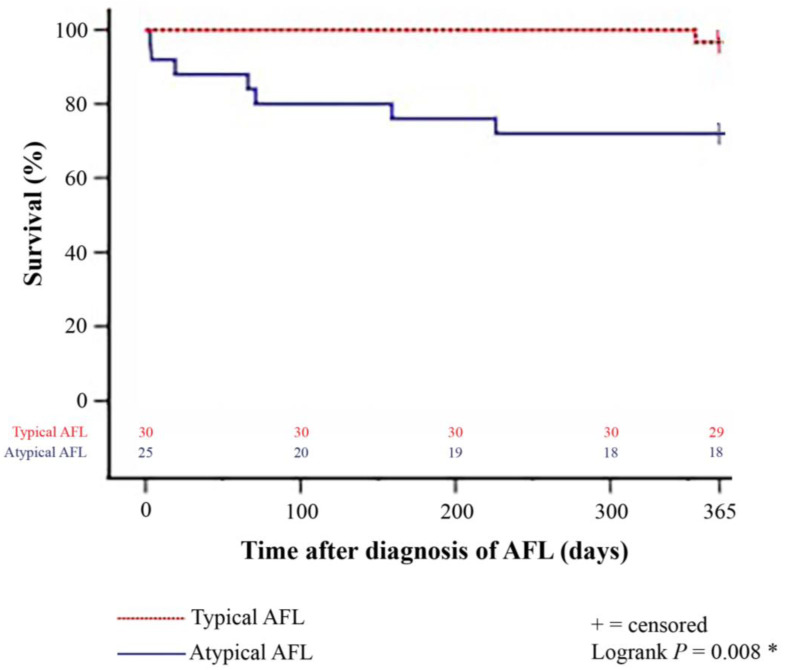
One-year survival after diagnosis of late-onset post-transplant AFL in patients with typical and atypical AFL (Kaplan–Meier estimator). Patients with atypical late-onset post-transplant AFL showed a statistically significant inferior 1-year post-transplant survival (18 of 25 [72.0%]) in comparison to patients with typical late-onset post-transplant AFL (29 of 30 [96.7%]; *p* = 0.008). AFL = atrial flutter; * = statistically significant (*p* < 0.050).

A total of 8 patients (14.5%) deceased within one year after diagnosis of late-onset post-transplant AFL. Patients with atypical AFL showed a significantly higher all-cause 1-year mortality than patients with typical AFL (7 of 25 [28.0%] versus 1 of 30 [3.3%]; *p* = 0.010). The Kaplan–Meier estimator further showed a statistically significant inferior 1-year survival after diagnosis of late-onset post-transplant AFL in patients with atypical AFL (18 of 25 [72.0%]) in comparison to patients with typical AFL (29 of 30 [96.7%], *p* = 0.008). The Kaplan–Meier survival curves are displayed in Figure 2.

Stratified by causes of death, patients with atypical AFL also had a significantly higher rate of graft failure within one year after diagnosis of late-onset post-transplant AFL (4 of 25 [16.0%] versus 0 of 30 [0.0%]; *p* = 0.023). Mortality and causes of death within one year after diagnosis of late-onset AFL after HTX are presented in Table 8.

### 3.7. Sensitivity Analysis

Given the long study period, we performed a sensitivity analysis with a subgroup of HTX recipients who received tacrolimus and mycophenolate mofetil as immunosuppressive drug therapy [292 of 639 HTX recipients (45.7%)] in order to investigate a possible era effect and to examine the robustness of our results. This analysis showed similar findings supporting the robustness of our results and reducing the likelihood of a potential era effect.

## 4. Discussion

### 4.1. Frequency and Risk Factors of Late-Onset Post-Transplant Atrial Flutter

Late-onset post-transplant AFL is a common complication late after HTX and plays an important role in the clinical management of HTX recipients [20]. Previous studies reported late-onset post-transplant AFL rates ranging from 8.0% to 11.5% [25,26,28,29]. Pavri and colleagues [28] published a late-onset post-transplant AFL rate of 8.0% (7 of 88) and Anselmino and colleagues [29] found a late-onset post-transplant AFL rate of 11.5% (42 of 364). This is in line with our late-onset post-transplant AFL rate of 8.6% (55 of 639). Regarding risk factors, the use of biatrial anastomosis has been associated with late-onset post-transplant AFL [20,22,23]. In HTX recipients with biatrial anastomosis, both donor atria are joined with most of the recipient atria resulting in enlarged atrial cavities with disruption of atrial anatomy and two long atrial anastomoses with plenty of potentially proarrhythmic scar tissue and substrate for a macro-reentry [17,20,21,22,23,24,25,26]. In addition, there is also a higher rate of moderate to severe tricuspid regurgitation which has also been linked to late-onset post-transplant AFL [20,22,23,29]. Likewise, we observed a higher percentage of biatrial anastomosis and moderate to severe tricuspid regurgitation in patients with late-onset post-transplant AFL.

Another important finding of this study is the fact that patients with late-onset post-transplant AFL had a younger recipient age at the time of HTX. As the development of late-onset post-transplant AFL takes many years, older HTX recipients may not survive for such a long period and long-term survivors after HTX have a reported younger recipient age [24,25,29,30]. The median interval from HTX to the initial diagnosis of late-onset post-transplant AFL was around nine years in our study. Furthermore, lower HTX recipient age has been associated with graft rejection which in turn has been linked to late-onset post-transplant AFL [20,21,22,23,24,25,31,32]. Younger HTX recipients have a stronger immune system with increased alloreactivity, but adherence to prescribed medications and recommended lifestyle habits are less strict [31,32,33]. In this light, the above-mentioned risk factors acutely emphasize the aspects of time, altered anatomy, and proarrhythmic substrate in the development of late-onset post-transplant AFL.

### 4.2. Clinical Findings of Typical and Atypical Late-Onset Post-Transplant Atrial Flutter

To our knowledge, this is the largest study of HTX recipients with in-depth data analysis between patients with typical and atypical late-onset post-transplant AFL. Out of 55 HTX recipients with late-onset post-transplant AFL, 30 patients had typical AFL (54.5%) and 25 patients had atypical AFL (45.5%). Typical AFL was more common in male HTX recipients which is also the case in the general population [34,35]. Patients with atypical AFL showed a higher percentage of arterial hypertension, reduced right and left ventricular function (systolic, diastolic, and longitudinal function), as well as mitral regurgitation which may be the result of chronic changes and underlying cardiac allograft vasculopathy [20,21,22,23,24,25]. This is in line with our findings that HTX recipients with atypical AFL had a significantly higher percentage of coronary artery disease with requirement for coronary stent implantation and a higher all-cause 1-year mortality along with a higher rate of graft failure after diagnosis of AFL. Matters are complicated further by the fact that more than a quarter of HTX recipients with atypical AFL were asymptomatic on presentation, and asymptomatic AFL can remain unnoticed for weeks or even months until it is incidentally found on routine resting 12-lead ECG or on routine 24-h Holter monitoring [34,35].

In contrast, most HTX recipients with typical AFL were symptomatic in our study. The majority of these patients had palpitations which might result from a significantly higher percentage of AFL with 2:1 atrioventricular conduction in comparison to patients with atypical AFL. Palpitations as a main symptom of HTX recipients with typical AFL were also reported in smaller studies with AFL after HTX [24,36,37]. Furthermore, HTX recipients with typical AFL had a significantly higher percentage of acute graft rejection. This may explain why previous studies were inconclusive about an association between graft rejection and post-transplant AFL in general [20,21,22,23,24,25,26]. Given these differences between patients with typical and atypical late-onset post-transplant AFL, we particularly recommend myocardial biopsy to exclude acute graft rejection in HTX recipients with typical AFL and cardiac catheterization to rule out presence of acute myocardial ischemia in HTX recipients with atypical AFL.

### 4.3. Treatment and Clinical Outcomes of Patients with Late-Onset Post-Transplant Atrial Flutter

Clinical management of HTX recipients with late-onset post-transplant AFL involves electrical cardioversion, pharmacological cardioversion, catheter ablation, and in some cases conservative treatment due to spontaneous conversion of AFL [20,21,22,23,24,25,26,36,37,38,39,40,41,42]. Initial standard treatment of patients with new-onset AFL after HTX predominantly consists of electrical or pharmacological cardioversion [20,21,22,23,24,25,26]. Likewise, the majority of patients with new-onset AFL after HTX received electrical (70.9%) or pharmacological cardioversion (16.4%) in our study. Although electrical or pharmacological cardioversion may be effective tools to quickly terminate AFL, recurrence of AFL after HTX is common, especially in patients with cardiac remodeling and graft dysfunction [20,21,22,23,24,25,43]. This is in line with our findings, as we also observed a high recurrence rate of late-onset AFL after HTX (83.6%) indicating the need for a safe and effective treatment for AFL after HTX.

During the last two decades, treatment and clinical management of HTX recipients with late-onset post-transplant AFL has changed towards a more invasive approach focusing on radiofrequency catheter ablation with three-dimensional electroanatomical mapping systems in order to better understand the underlying mechanisms of post-transplant AFL [20,21,22,23,24,25,26,36,37,38,39,40,41,42]. Taylor and colleagues [25] reported that they treated post-transplant AFL with electrical or pharmacological cardioversion prior to 2002 and started performing catheter ablation for post-transplant AFL hereafter. Several studies demonstrated that catheter ablation is a safe and effective treatment for HTX recipients with post-transplant AFL although the distorted atrial anatomy of the transplanted heart may be challenging regarding optimal catheter placement for ablation of AFL [20,21,22,23,24,25,26,36,37,38,39,40,41,42].

Rodríguez-Entem and colleagues [24] performed successful catheter ablation of the cavotricuspid isthmus in 12 of 13 HTX recipients (92.3%) with post-transplant AFL. They observed a recurrence of post-transplant AFL in 3 of 12 HTX recipients (25.0%) who underwent repeat catheter ablation during a mean follow-up of 24 ± 17 months [24]. Vaseghi and colleagues [26] performed catheter ablation of the cavotricuspid isthmus in 14 HTX recipients with typical post-transplant AFL and reported recurrence of AFL in two patients (14.3%) who required a second catheter ablation [26]. Similar results were reported by Mouhoub and colleagues [40] who performed catheter ablation of the cavotricuspid isthmus in 28 of 30 HTX recipients (93.3%) with typical post-transplant AFL as well as catheter ablation of atypical post-transplant AFL in 2 of 30 HTX recipients (6.7%). They reported a primary catheter ablation success in 28 of 30 HTX recipients (93.3%) [40]. The largest study on catheter ablation of post-transplant AFL so far was published by Taylor and colleagues [25] who performed catheter ablation of the cavotricuspid isthmus in 26 of 32 HTX recipients (81.2%) with typical post-transplant AFL as well as catheter ablation of atypical post-transplant AFL in 6 of 32 HTX recipients (18.8%). In this case, 8 of the 32 patients (25.0%) underwent repeat catheter ablation [25].

In our study, 20 HTX recipients with recurrence of late-onset post-transplant AFL received catheter ablation (13 patients with typical AFL and 7 patients with atypical AFL). Only 2 of these 20 HTX recipients (10.0%) had a further episode of post-transplant AFL within one year after catheter ablation. In comparison to HTX recipients without catheter ablation after recurrence of late-onset post-transplant AFL, HTX recipients with catheter ablation after recurrence of late-onset post-transplant AFL showed a significantly lower 1-year rate of further episodes of post-transplant AFL (*p* = 0.003). These findings are of high clinical relevance as there is a high risk of AFL recurrence in patients after HTX, and standard treatment of patients with AFL after HTX still consists of electrical or pharmacological cardioversion. Therefore, given the distinct cardiovascular risk profile of HTX recipients with late-onset post-transplant AFL, catheter ablation represents a viable option for HTX recipients in order to avoid further episodes of post-transplant AFL.

### 4.4. Study Limitations

The results of our study were derived from a large single-center registry (Heidelberg HTX Registry) including the highly detailed data of 639 patients who received HTX at the Heidelberg Heart Center. In awareness of the known limitations of such a study design, our findings should be interpreted carefully and within the context of the existing literature. However, we would like to emphasize that our study was comparable to multicenter studies in sample size and our patients received standardized treatment and follow-up, reducing the likelihood of selection bias and potential confounders [11,12,13,14,15,16,17,18,19].

Long-term follow-up is essential to detect late-onset post-transplant AFL. We therefore decided to analyze patients who received HTX at the Heidelberg Heart Center between 1989 and 2019 providing a minimum follow-up of two years after HTX. As a consequence of the long study period of more than 30 years, a possible era effect due to changes in surgical and medical care may have influenced our findings. In order to investigate a possible era effect, we performed a sensitivity analysis with HTX recipients who received tacrolimus and mycophenolate mofetil, since tacrolimus replaced cyclosporine A as the main immunosuppressive agent from 2006 onward. This analysis showed similar results supporting the robustness of our findings [11,12,13,14,15,16,17,18,19].

Assessment of late-onset post-transplant AFL was based upon all available source files including resting 12-lead ECGs, monitor-telemetry, 24-h Holter monitoring, and EPS data in the case of performed ablation. The type of AFL was diagnosed on 12-lead ECG criteria [27] but an atypical ECG pattern could not exclude CTI-dependent macro-reentrant tachycardia. As EPS was not performed in all patients with late-onset AFL after HTX, we cannot rule out that some patients with an ECG pattern of atypical AFL who did not undergo catheter ablation might have been misclassified. It is also possible that asymptomatic episodes of AFL could have been missed. However, as most HTX recipients with AFL were symptomatic and patients after HTX were routinely followed-up with resting 12-lead ECG and 24-h Holter monitoring, it is very unlikely that a significant number of HTX recipients with AFL were undetected. In addition, our findings should be interpreted as hypothesis-generating, especially in the context of risk factors for late-onset post-transplant AFL and mortality after HTX, because multiple factors can influence these outcomes. Furthermore, the long-term effects of catheter ablation in HTX recipients with AFL after HTX remain unknown and require further investigation, preferably in the form of large multicenter trials.

## 5. Conclusions

Post-transplant AFL is a common and clinically relevant cardiac arrhythmia late after HTX. About one out of ten HTX recipients suffered from it and the median interval from HTX until the initial diagnosis of late-onset post-transplant AFL was around nine years. Patients with late-onset post-transplant AFL had a lower recipient age at HTX, received more biatrial anastomosis, and showed a higher rate of moderate or severe tricuspid regurgitation, all underlining the aspects of time, altered anatomy, and proarrhythmic substrate in the development of late-onset post-transplant AFL. Regarding the differences between typical and atypical AFL, typical AFL was associated with symptomatic palpitations and acute graft rejection, while atypical AFL was common in asymptomatic HTX recipients with coronary artery disease and requirement for coronary stent implantation. Impaired right-sided heart function was found in more than half of HTX recipients with late-onset post-transplant AFL, but a significant number of patients with atypical AFL also showed a reduced left-sided heart function and had a higher all-cause 1-year mortality along with a higher rate of graft failure after diagnosis of AFL. Initial standard treatment of patients with new-onset AFL after HTX mainly consists of electrical or pharmacological cardioversion, whereas catheter ablation is often only used in cases of recurrence. However, since most patients with late-onset AFL after HTX suffer from recurrence of AFL and the recurrence rate of AFL after catheter ablation is significantly lower, catheter ablation may not only represent a viable option for HTX recipients with recurrence of post-transplant AFL but also for HTX recipients with new-onset post-transplant AFL, especially in the light of the risk profile and vulnerability of these patients.

## Figures and Tables

**Table 1:** Demographics-stratified by late-onset AFL after HTX.

Parameter	All (*n* = 639)	No AFL after HTX (*n* = 584)	AFL after HTX (*n* = 55)	Difference	95% CI	*p*-Value
**Recipient data**						
Age (years), mean ± SD	52.1 ± 10.3	52.4 ± 10.2	48.8 ± 11.2	3.6	0.5–6.7	0.028 *
Male sex, *n* (%)	498 (77.9%)	456 (78.1%)	42 (76.4%)	1.7%	−10.0–13.4%	0.769
BMI (kg/m^2^), mean ± SD	24.9 ± 4.0	25.0 ± 4.0	24.4 ± 3.5	0.6	−0.4–1.6	0.285
Coronary artery disease ^†^	259 (40.5%)	240 (41.1%)	19 (34.5%)	6.6%	−6.6–19.8%	0.344
Arterial hypertension, *n* (%)	350 (54.8%)	321 (55.0%)	29 (52.7%)	2.3%	−11.5–16.1%	0.750
Dyslipidemia, *n* (%)	406 (63.5%)	377 (64.6%)	29 (52.7%)	11.9%	−1.9–25.7%	0.081
Diabetes mellitus, *n* (%)	215 (33.6%)	199 (34.1%)	16 (29.1%)	5.0%	−7.6–17.6%	0.455
Peripheral artery disease, *n* (%)	84 (13.1%)	78 (13.4%)	6 (10.9%)	2.5%	−6.2–11.2%	0.608
COPD, *n* (%)	155 (24.3%)	146 (25.0%)	9 (16.4%)	8.6%	−1.8–19.0%	0.153
History of smoking, *n* (%)	387 (60.6%)	355 (60.8%)	32 (58.2%)	2.6%	−11.0–16.2%	0.705
Pack years (py), mean ± SD	12.4 ± 14.3	12.6 ± 14.3	10.5 ± 13.9	2.1	−1.8–6.0	0.299
Renal insufficiency ^, *n* (%)	368 (57.6%)	335 (57.4%)	33 (60.0%)	2.6%	−10.9–16.1%	0.705
eGFR (mL/min/1.73 m^2^), mean ± SD	60.3 ± 21.7	60.1 ± 21.4	61.5 ± 24.5	1.4	−5.3–8.1	0.696
**Previous open-heart surgery**						
Overall open-heart surgery, *n* (%)	190 (29.7%)	176 (30.1%)	14 (25.5%)	4.6%	−7.5–16.7%	0.468
CABG surgery, *n* (%)	78 (12.2%)	72 (12.3%)	6 (10.9%)	1.4%	−7.2–10.0%	0.758
Other surgery °, *n* (%)	71 (11.1%)	68 (11.6%)	3 (5.5%)	6.1%	−0.4–12.6%	0.163
VAD surgery, *n* (%)	55 (8.6%)	50 (8.6%)	5 (9.1%)	0.5%	−7.4–8.4%	0.894
**Principal diagnosis for HTX**						
Ischemic CMP, *n* (%)	209 (32.7%)	192 (32.9%)	17 (30.9%)	2.0%	−10.8–14.8%	0.766
Non-ischemic CMP, *n* (%)	339 (53.1%)	307 (52.6%)	32 (58.2%)	5.6%	−8.0–19.2%	0.425
Valvular heart disease, *n* (%)	34 (5.3%)	32 (5.5%)	2 (3.6%)	1.9%	−3.4–7.2%	0.560
Cardiac amyloidosis, *n* (%)	57 (8.9%)	53 (9.1%)	4 (7.3%)	1.8%	−5.5–9.1%	0.654
**Donor data**						
Age (years), mean ± SD	41.0 ± 13.4	41.0 ± 13.5	41.5 ± 12.9	0.5	−3.1–4.1	0.762
Male sex, *n* (%)	278 (43.5%)	248 (42.5%)	30 (54.5%)	12.0%	−1.8–25.8%	0.084
BMI (kg/m^2^), mean ± SD	24.8 ± 4.1	24.8 ± 4.1	25.0 ± 3.8	0.2	−0.9–1.3	0.666
**Transplant sex mismatch**						
Mismatch, *n* (%)	283 (44.3%)	261 (44.7%)	22 (40.0%)	4.7%	−8.9–18.3%	0.503
Donor (m) to recipient (f), *n* (%)	31 (4.9%)	26 (4.5%)	5 (9.1%)	4.6%	−3.2–12.4%	0.126
Donor (f) to recipient (m), *n* (%)	252 (39.4%)	235 (40.2%)	17 (30.9%)	9.3%	−3.5–22.1%	0.176
**Perioperative data**						
Ischemic time (min), mean ± SD	223.4 ± 68.4	224.7 ± 68.1	209.4 ± 71.2	15.3	−4.3–34.9	0.131
Biatrial anastomosis, *n* (%)	164 (25.7%)	140 (24.0%)	24 (43.6%)	19.6%	6.1–33.1%	0.001 *
Bicaval anastomosis, *n* (%)	475 (74.3%)	444 (76.0%)	31 (56.4%)	19.6%	6.1–33.1%	0.001 *

AFL = atrial flutter; BMI = body mass index; CABG = coronary artery bypass graft; CI = confidence interval; CMP = cardiomyopathy; COPD = chronic obstructive pulmonary disease; f = female; eGFR = estimated glomerular filtration rate; HTX = heart transplantation; m = male; *n* = number; py = pack year; SD = standard deviation; VAD = ventricular assist device; ^†^ = presence of coronary artery disease before HTX; ^ = eGFR < 60 mL/min/1.73 m^2^; ° = congenital, valvular or ventricular surgery; * = statistically significant (*p* < 0.050).

**Table 2:** Demographics-stratified by typical and atypical late-onset AFL after HTX.

Parameter	AFL after HTX (*n* = 55)	Typical AFL after HTX (*n* = 30)	Atypical AFL after HTX (*n* = 25)	Difference	95% CI	*p*-Value
**Recipient data**						
Age (years), mean ± SD	48.8 ± 11.2	47.9 ± 12.6	50.0 ± 9.3	2.1	−3.7–7.9	0.490
Male sex, *n* (%)	42 (76.4%)	26 (86.7%)	16 (64.0%)	22.7%	0.3–45.1%	0.049 *
BMI (kg/m^2^), mean ± SD	24.4 ± 3.5	24.4 ± 3.0	24.5 ± 4.1	0.1	−1.8–2.0	0.978
Coronary artery disease ^†^	19 (34.5%)	9 (30.0%)	10 (40.0%)	10.0%	−15.3–35.3%	0.437
Arterial hypertension, *n* (%)	29 (52.7%)	12 (40.0%)	17 (68.0%)	28.0%	2.7–53.3%	0.038 *
Dyslipidemia, *n* (%)	29 (52.7%)	13 (43.3%)	16 (64.0%)	20.7%	−5.2–46.6%	0.126
Diabetes mellitus, *n* (%)	16 (29.1%)	8 (26.7%)	8 (32.0%)	5.3%	−18.9–29.5%	0.665
Peripheral artery disease, *n* (%)	6 (10.9%)	5 (16.7%)	1 (4.0%)	12.7%	−2.7–28.1%	0.134
COPD, *n* (%)	9 (16.4%)	4 (13.3%)	5 (20.0%)	6.7%	−13.1–26.5%	0.506
History of smoking, *n* (%)	32 (58.2%)	19 (63.3%)	13 (52.0%)	11.3%	−14.8–37.4%	0.396
Pack years (py), mean ± SD	10.5 ± 13.9	10.0 ± 10.3	11.2 ± 17.5	1.2	−6.6–9.0	0.764
Renal insufficiency ^, *n* (%)	33 (60.0%)	19 (63.3%)	14 (56.0%)	7.3%	−18.7–33.3%	0.580
eGFR (mL/min/1.73 m^2^), mean ± SD	61.5 ± 24.5	66.0 ± 29.2	56.0 ± 16.3	10.0	−2.2–22.2	0.116
**Previous open-heart surgery**						
Overall open-heart surgery, *n* (%)	14 (25.5%)	9 (30.0%)	5 (20.0%)	10.0%	−12.7–32.7%	0.397
CABG surgery, *n* (%)	6 (10.9%)	3 (10.0%)	3 (12.0%)	2.0%	−14.7–18.7%	0.813
Other surgery °, *n* (%)	3 (5.5%)	3 (10.0%)	0 (0.0%)	10.0%	−0.7–20.7%	0.104
VAD surgery, *n* (%)	5 (9.1%)	3 (10.0%)	2 (8.0%)	2.0%	−13.1–17.1%	0.797
**Principal diagnosis for HTX**						
Ischemic CMP, *n* (%)	17 (30.9%)	8 (26.7%)	9 (36.0%)	9.3%	−15.3–33.9%	0.456
Non-ischemic CMP, *n* (%)	32 (58.2%)	18 (60.0%)	14 (56.0%)	4.0%	−22.2–30.2%	0.765
Valvular heart disease, *n* (%)	2 (3.6%)	2 (6.7%)	0 (0.0%)	6.7%	−2.2–15.6%	0.188
Cardiac amyloidosis, *n* (%)	4 (7.3%)	2 (6.7%)	2 (8.0%)	1.3%	−12.6–15.2%	0.850
**Donor data**						
Age (years), mean ± SD	41.5 ± 12.9	40.6 ± 13.6	42.6 ± 12.3	2.0	−4.8–8.8	0.569
Male sex, *n* (%)	30 (54.5%)	19 (63.3%)	11 (44.0%)	19.3%	−6.7–45.3%	0.152
BMI (kg/m^2^), mean ± SD	25.0 ± 3.8	25.6 ± 3.8	24.4 ± 3.8	1.2	−0.8–3.2	0.231
**Transplant sex mismatch**						
Mismatch, *n* (%)	22 (40.0%)	11 (36.7%)	11 (44.0%)	7.3%	−18.7–33.3%	0.580
Donor (m) to recipient (f), *n* (%)	5 (9.1%)	2 (6.7%)	3 (12.0%)	5.3%	−10.3–20.9%	0.493
Donor (f) to recipient (m), *n* (%)	17 (30.9%)	9 (30.0%)	8 (32.0%)	2.0%	−22.6–26.6%	0.873
**Perioperative data**						
Ischemic time (min), mean ± SD	209.4 ± 71.2	212.8 ± 78.8	205.4 ± 62.2	7.4	−29.9–44.7	0.697
Biatrial anastomosis, *n* (%)	24 (43.6%)	12 (40.0%)	12 (48.0%)	8.0%	−18.3–34.3%	0.551
Bicaval anastomosis, *n* (%)	31 (56.4%)	18 (60.0%)	13 (52.0%)	8.0%	−18.3–34.3%	0.551

AFL = atrial flutter; BMI = body mass index; CABG = coronary artery bypass graft; CI = confidence interval; CMP = cardiomyopathy; COPD = chronic obstructive pulmonary disease; f = female; eGFR = estimated glomerular filtration rate; HTX = heart transplantation; m = male; *n* = number; py = pack year; SD = standard deviation; VAD = ventricular assist device; ^†^ = presence of coronary artery disease before HTX; ^ = eGFR < 60 mL/min/1.73 m^2^; ° = congenital, valvular or ventricular surgery; * = statistically significant (*p* < 0.050).

**Table 3:** Medications-stratified by late-onset AFL after HTX.

Parameter	All (*n* = 639)	No AFL after HTX (*n* = 584)	AFL after HTX (*n* = 55)	Difference	95% CI	*p*-Value
**Immunosuppressive drug therapy**						
Cyclosporine A, *n* (%)	347 (54.3%)	312 (53.4%)	35 (63.6%)	10.2%	−3.2–23.6%	0.146
Tacrolimus, *n* (%)	292 (45.7%)	272 (46.6%)	20 (36.4%)	10.2%	−3.2–23.6%	0.146
Azathioprine, *n* (%)	267 (41.8%)	240 (41.1%)	27 (49.1%)	8.0%	−5.8–21.8%	0.250
Mycophenolate mofetil, *n* (%)	372 (58.2%)	344 (58.9%)	28 (50.9%)	8.0%	−5.8–21.8%	0.250
Steroids, *n* (%)	639 (100.0%)	584 (100.0%)	55 (100.0%)	0.0%	n. a.	n. a.
**Concomitant medications**						
ASA, *n* (%)	68 (10.6%)	60 (10.3%)	8 (14.5%)	4.2%	−5.4–13.8%	0.326
Beta blocker, *n* (%)	114 (17.8%)	106 (18.2%)	8 (14.5%)	3.7%	−6.1–13.5%	0.504
Ivabradine, *n* (%)	61 (9.5%)	58 (9.9%)	3 (5.5%)	4.4%	−2.1–10.9%	0.280
Calcium channel blocker, *n* (%)	171 (26.8%)	161 (27.6%)	10 (18.2%)	9.4%	−1.4–20.2%	0.133
ACE inhibitor/ARB, *n* (%)	278 (43.5%)	250 (42.8%)	28 (50.9%)	8.1%	−5.7–21.9%	0.247
Diuretic, *n* (%)	639 (100.0%)	584 (100.0%)	55 (100.0%)	0.0%	n. a.	n. a.
Statin, *n* (%)	254 (39.7%)	232 (39.7%)	22 (40.0%)	0.3%	−13.3–13.9%	0.968
Gastric protection ^†^, *n* (%)	639 (100.0%)	584 (100.0%)	55 (100.0%)	0.0%	n. a.	n. a.

ACE inhibitor = angiotensin-converting-enzyme inhibitor; AFL = atrial flutter; ARB = angiotensin II receptor blocker; ASA = acetylsalicylic acid; CI = confidence interval; HTX = heart transplantation; *n* = number; n. a. = not applicable; ^†^ = gastric protection defined as proton pump inhibitor (PPI) or histamine receptor (H2) blocker.

**Table 4:** Medications-stratified by typical and atypical late-onset AFL after HTX.

Parameter	AFLafter HTX (*n* = 55)	Typical AFL after HTX (*n* = 30)	Atypical AFL after HTX (*n* = 25)	Difference	95% CI	*p*-Value
**Immunosuppressive drug therapy**						
Cyclosporine A, *n* (%)	35 (63.6%)	18 (60.0%)	17 (68.0%)	8.0%	−17.3–33.3%	0.539
Tacrolimus, *n* (%)	20 (36.4%)	12 (40.0%)	8 (32.0%)	8.0%	−17.3–33.3%	0.539
Azathioprine, *n* (%)	27 (49.1%)	13 (43.3%)	14 (56.0%)	12.7%	−13.6–39.0%	0.349
Mycophenolate mofetil, *n* (%)	28 (50.9%)	17 (56.7%)	11 (44.0%)	12.7%	−13.6–39.0%	0.349
Steroids, *n* (%)	55 (100.0%)	30 (100.0%)	25 (100.0%)	0.0%	n. a.	n. a.
**Concomitant medications**						
ASA, *n* (%)	8 (14.5%)	2 (6.7%)	6 (24.0%)	17.3%	−1.7–36.3%	0.069
Beta blocker, *n* (%)	8 (14.5%)	5 (16.7%)	3 (12.0%)	4.7%	−13.7–23.1%	0.625
Ivabradine, *n* (%)	3 (5.5%)	2 (6.7%)	1 (4.0%)	2.7%	−9.1–14.5%	0.665
Calcium channel blocker, *n* (%)	10 (18.2%)	7 (23.3%)	3 (12.0%)	11.3%	−8.5–31.1%	0.278
ACE inhibitor/ARB, *n* (%)	28 (50.9%)	17 (56.7%)	11 (44.0%)	12.7%	−13.6–39.0%	0.349
Diuretic, *n* (%)	55 (100.0%)	30 (100.0%)	25 (100.0%)	0.0%	n. a.	n. a.
Statin, *n* (%)	22 (40.0%)	15 (50.0%)	7 (28.0%)	22.0%	−3.1–47.1%	0.097
Gastric protection ^†^, *n* (%)	55 (100.0%)	30 (100.0%)	25 (100.0%)	0.0%	n. a.	n. a.

ACE inhibitor = angiotensin-converting-enzyme inhibitor; AFL = atrial flutter; ARB = angiotensin II receptor blocker; ASA = acetylsalicylic acid; CI = confidence interval; HTX = heart transplantation; *n* = number; n. a. = not applicable; ^†^ = gastric protection defined as proton pump inhibitor (PPI) or histamine receptor (H2) blocker.

**Table 5:** Clinical presentation and findings of patients with late-onset AFL after HTX.

Parameter	AFL after HTX (*n* = 55)	Typical AFL after HTX (*n* = 30)	Atypical AFL after HTX (*n* = 25)	Difference	95% CI	*p*-Value
**Symptomatic**						
Symptomatic finding, *n* (%)	46 (83.6%)	28 (93.3%)	18 (72.0%)	21.3%	1.6–41.0%	0.033 *
Palpitations, *n* (%)	41 (74.5%)	26 (86.7%)	15 (60.0%)	26.7%	4.0–49.4%	0.024 *
Dizziness/lightheadedness, *n* (%)	29 (52.7%)	15 (50.0%)	14 (56.0%)	6.0%	−20.4–32.4%	0.657
Fainting/syncope, *n* (%)	4 (7.3%)	1 (3.3%)	3 (12.0%)	8.7%	−5.6–23.0%	0.218
Fatigue/exercise intolerance, *n* (%)	33 (60.0%)	19 (63.3%)	14 (56.0%)	7.3%	−18.7–33.3%	0.580
Shortness of breath, *n* (%)	32 (58.2%)	18 (60.0%)	14 (56.0%)	4.0%	−22.2–30.2%	0.765
Chest Pain, *n* (%)	9 (16.4%)	2 (6.7%)	7 (28.0%)	21.3%	1.6–41.0%	0.033 *
Anxiety, *n* (%)	33 (60.0%)	19 (63.3%)	14 (56.0%)	7.3%	−18.7–33.3%	0.580
Peripheral edema, *n* (%)	22 (40.0%)	8 (26.7%)	14 (56.0%)	29.3%	4.2–54.4%	0.027 *
**Asymptomatic**						
Asymptomatic finding, *n* (%)	9 (16.4%)	2 (6.7%)	7 (28.0%)	21.3%	1.6–41.0%	0.033 *
Routine resting 12-lead ECG, *n* (%)	7 (12.7%)	1 (3.3%)	6 (24.0%)	20.7%	2.8–38.6%	0.022 *
Routine 24-h Holter monitoring, *n* (%)	2 (3.6%)	1 (3.3%)	1 (4.0%)	0.7%	−9.3–10.7%	0.895
**Atrioventricular conduction**						
AFL with 2:1 conduction, *n* (%)	29 (52.7%)	20 (66.7%)	9 (36.0%)	30.7%	5.4–56.0%	0.023 *
AFL with 3:1 conduction, *n* (%)	15 (27.3%)	7 (23.3%)	8 (32.0%)	8.7%	−15.0–32.4%	0.472
AFL with 4:1 conduction, *n* (%)	7 (12.7%)	2 (6.7%)	5 (20.0%)	13.3%	−4.7–31.3%	0.140
AFL with variable conduction, *n* (%)	4 (7.3%)	1 (3.3%)	3 (12.0%)	8.7%	−5.6–23.0%	0.218
**Clinical findings**						
Coronary artery disease, *n* (%)	20 (36.4%)	7 (23.3%)	13 (52.0%)	28.7%	4.0–53.4%	0.028 *
Coronary stent implantation, *n* (%)	11 (20.0%)	3 (10.0%)	8 (32.0%)	22.0%	0.8–43.2%	0.042 *
Acute graft rejection, *n* (%)	18 (32.7%)	14 (46.7%)	4 (16.0%)	30.7%	7.8–53.6%	0.016 *
Acute infection, *n* (%)	11 (20.0%)	3 (10.0%)	8 (32.0%)	22.0%	0.8–43.2%	0.042 *
Hyperthyroidism, *n* (%)	1 (1.8%)	1 (3.3%)	0 (0.0%)	3.3%	−3.1–9.7%	0.357
Recent surgery (≤30 days), *n* (%)	3 (5.5%)	2 (6.7%)	1 (4.0%)	2.7%	−9.1–14.5%	0.665
Electrolyte imbalance, *n* (%)	4 (7.3%)	1 (3.3%)	3 (12.0%)	8.7%	−5.6–23.0%	0.218
Chronic hemodialysis, *n* (%)	11 (20.0%)	3 (10.0%)	8 (32.0%)	22.0%	0.8–43.2%	0.042 *

AFL = atrial flutter; CI = confidence interval; ECG = electrocardiogram; h = hour; HTX = heart transplantation; *n* = number; * = statistically significant (*p* < 0.050).

**Table 6:** Echocardiographic features of patients with late-onset AFL after HTX.

Parameter	AFL after HTX (*n* = 55)	Typical AFL after HTX (*n* = 30)	Atypical AFL after HTX (*n* = 25)	Difference	95% CI	*p*-Value
**End-diastolic diameter**						
Normal RA (<35 mm), *n* (%)	10 (18.2%)	7 (23.3%)	3 (12.0%)	11.3%	−8.5–31.1%	0.278
RA diameter (mm), mean ± SD	37.6 ± 4.6	36.8 ± 3.9	38.6 ± 5.2	1.8	−0.7–4.3	0.143
Normal RV (<30 mm), *n* (%)	16 (29.1%)	11 (36.7%)	5 (20.0%)	16.7%	−6.6–40.0%	0.175
RV diameter (mm), mean ± SD	36.6 ± 6.4	36.1 ± 7.0	37.2 ± 5.8	1.1	−2.3–4.5	0.539
Normal LA (<40 mm), *n* (%)	15 (27.3%)	12 (40.0%)	3 (12.0%)	28.0%	6.3–49.7%	0.020 *
LA diameter (mm), mean ± SD	43.9 ± 5.5	42.2 ± 4.6	46.0 ± 5.8	3.8	1.0–6.6	0.011 *
Normal LV (<55 mm), *n* (%)	41 (74.5%)	26 (86.7%)	15 (60.0%)	26.7%	3.9–49.5%	0.024 *
LV diameter (mm), mean ± SD	50.8 ± 5.5	49.2 ± 4.6	52.8 ± 5.9	3.6	0.7–6.5	0.019 *
**RV function**						
Normal, *n* (%)	26 (47.3%)	18 (60.0%)	8 (32.0%)	28.0%	2.7–53.3%	0.038 *
Reduced, *n* (%)	29 (52.7%)	12 (40.0%)	17 (68.0%)	28.0%	2.7–53.3%	0.038 *
Mild, *n* (%)	17 (30.9%)	4 (13.3%)	13 (52.0%)	38.7%		
Moderate, *n* (%)	3 (5.5%)	3 (10.0%)	0 (0.0%)	10.0%		
Severe, *n* (%)	9 (16.4%)	5 (16.7%)	4 (16.0%)	0.7%		
**LV function**						
Normal, *n* (%)	35 (63.6%)	25 (83.3%)	10 (40.0%)	43.3%	19.9–66.7%	0.001 *
Reduced, *n* (%)	20 (36.4%)	5 (16.7%)	15 (60.0%)	43.3%	19.9–66.7%	0.001 *
Mild, *n* (%)	10 (18.2%)	2 (6.7%)	8 (32.0%)	25.3%		
Moderate, *n* (%)	6 (10.9%)	2 (6.7%)	4 (16.0%)	9.3%		
Severe, *n* (%)	4 (7.3%)	1 (3.3%)	3 (12.0%)	8.7%		
**Longitudinal function**						
Normal TAPSE (≥17 mm), *n* (%)	31 (56.4%)	21 (70.0%)	10 (40.0%)	30.0%	4.8–55.2%	0.025 *
TAPSE (mm), mean ± SD	17.7 ± 4.7	19.0 ± 5.4	16.2 ± 3.1	2.8	0.5–5.1	0.021 *
Normal MAPSE (≥11 mm), *n* (%)	39 (70.9%)	27 (90.0%)	12 (48.0%)	42.0%	19.7–64.3%	0.001 *
MAPSE (mm), mean ± SD	13.4 ± 3.3	14.5 ± 2.5	12.1 ± 3.6	2.4	0.7–4.1	0.007 *
**Diastolic function**						
E/A ratio, mean ± SD	2.6 ± 0.9	2.3 ± 0.7	3.1 ± 1.0	0.8	0.3–1.3	0.002 *
E/e′ ratio, mean ± SD	11.1 ± 5.5	7.7 ± 3.4	15.1 ± 4.7	7.4	5.2–9.6	<0.001 *
DT-E (ms), mean ± SD	179.7 ± 36.2	201.7 ± 26.8	153.4 ± 27.6	48.3	33.8–62.8	<0.001 *
Systolic PAP (mmHg), mean ± SD	35.8 ± 10.6	32.6 ± 9.9	39.6 ± 10.2	7.0	1.7–12.3	0.013 *
RAP (mmHg), mean ± SD	10.0 ± 5.4	8.2 ± 4.3	12.2 ± 6.0	4.0	1.2–6.8	0.007 *
IVC, (mm), mean ± SD	21.5 ± 5.8	19.7 ± 5.0	23.6 ± 6.0	3.9	0.9–6.9	0.013 *
**Tricuspid regurgitation**						
No, *n* (%)	13 (23.6%)	8 (26.7%)	5 (20.0%)	6.7%	−15.6–29.0%	0.562
Yes, *n* (%)	42 (76.4%)	22 (73.3%)	20 (80.0%)	6.7%	−15.6–29.0%	0.562
Mild, *n* (%)	11 (20.0%)	6 (20.0%)	5 (20.0%)	0.0%		
Moderate, *n* (%)	15 (27.3%)	8 (26.7%)	7 (28.0%)	1.3%		
Severe, *n* (%)	16 (29.1%)	8 (26.7%)	8 (32.0%)	5.3%		
**Mitral regurgitation**						
No, *n* (%)	17 (30.9%)	13 (43.3%)	4 (16.0%)	27.3%	4.5–50.1%	0.029 *
Yes, *n* (%)	38 (69.1%)	17 (56.7%)	21 (84.0%)	27.3%	4.5–50.1%	0.029 *
Mild, *n* (%)	28 (50.9%)	14 (46.7%)	14 (56.0%)	9.3%		
Moderate, *n* (%)	6 (10.9%)	2 (6.7%)	4 (16.0%)	9.3%		
Severe, *n* (%)	4 (7.3%)	1 (3.3%)	3 (12.0%)	8.7%		

AFL = atrial flutter; CI = confidence interval; DT-E = deceleration time (DT) of the early diastolic mitral inflow peak (E); E/A = early diastolic mitral inflow peak velocity (E) to late diastolic mitral inflow peak velocity (A) ratio; E/e′ = early diastolic mitral inflow peak velocity (E) to early diastolic mitral annular velocity (e′) ratio; HTX = heart transplantation; IVC = inferior vena cava; LA = left atrium; LV = left ventricle; MAPSE = mitral annular plane systolic excursion; *n* = number; PAP = pulmonary artery pressure; RA = right atrium; RAP = right atrial pressure; RV = right ventricle; SD = standard deviation; TAPSE = tricuspid annular plane systolic excursion; * = statistically significant (*p* < 0.050).

**Table 7:** Treatment of patients with late-onset AFL after HTX.

Parameter	AFL after HTX (*n* = 55)	Typical AFL after HTX (*n* = 30)	Atypical AFL after HTX (*n* = 25)	Difference	95% CI	*p*-Value
**Initial occurrence of AFL after HTX**						
Initial occurrence of AFL, *n* (%)	55 (100.0%)	30 (100.0%)	25 (100.0%)	0.0%	n. a.	n. a.
Spontaneous conversion, *n* (%)	7 (12.7%)	2 (6.7%)	5 (20.0%)	13.3%	−4.7–31.3%	0.140
Electrical cardioversion, *n* (%)	39 (70.9%)	26 (86.7%)	13 (52.0%)	34.7%	11.6–57.8%	0.005 *
Pharmacological cardioversion, *n* (%)	9 (16.4%)	2 (6.7%)	7 (28.0%)	21.3%	1.6–41.0%	0.033 *
Catheter ablation, *n* (%)	0 (0.0%)	0 (0.0%)	0 (0.0%)	0.0%	n. a.	n. a.
**Recurrence of AFL after HTX**						
Recurrence of AFL, *n* (%)	46 (83.6%)	27 (90.0%)	19 (76.0%)	14.0%	−5.9–33.9%	0.162
Spontaneous conversion, *n* (%)	4 (7.3%)	1 (3.3%)	3 (12.0%)	8.7%	−5.6–23.0%	0.218
Electrical cardioversion, *n* (%)	15 (27.3%)	12 (40.0%)	3 (12.0%)	28.0%	6.3–49.7%	0.020 *
Pharmacological cardioversion, *n* (%)	7 (12.7%)	1 (3.3%)	6 (24.0%)	20.7%	2.8–38.6%	0.022 *
Catheter ablation, *n* (%)	20 (36.4%)	13 (43.3%)	7 (28.0%)	15.3%	−9.7–40.3%	0.239
**Further episodes of AFL after HTX**						
Further episodes of AFL, *n* (%)	19 (34.5%)	9 (30.0%)	10 (40.0%)	10.0%	−15.3–35.3%	0.437
Spontaneous conversion, *n* (%)	3 (5.5%)	1 (3.3%)	2 (8.0%)	4.7%	−7.7–17.1%	0.448
Electrical cardioversion, *n* (%)	5 (9.1%)	4 (13.3%)	1 (4.0%)	9.3%	−5.1–23.7%	0.231
Pharmacological cardioversion, *n* (%)	7 (12.7%)	1 (3.3%)	6 (24.0%)	20.7%	2.8–38.6%	0.022 *
Catheter ablation, *n* (%)	2 (3.6%)	2 (6.7%)	0 (0.0%)	6.7%	−2.2–15.6%	0.188
Repeat catheter ablation, *n* (%)	2 (3.6%)	1 (3.3%)	1 (4.0%)	0.7%	−9.3–10.7%	0.895

AFL = atrial flutter; CI = confidence interval; HTX = heart transplantation; *n* = number; n. a. = not applicable; * = statistically significant (*p* < 0.050).

**Table 8:** Causes of death within one year after diagnosis of late-onset AFL after HTX.

Parameter	AFL after HTX (*n* = 55)	Typical AFL after HTX (*n* = 30)	Atypical AFL after HTX (*n* = 25)	Difference	95% CI	*p*-Value
Graft failure, *n* (%)	4 (7.3%)	0 (0.0%)	4 (16.0%)	16.0%	1.6–30.4%	0.023 *
Acute rejection, *n* (%)	0 (0.0%)	0 (0.0%)	0 (0.0%)	0.0%	n. a.	n. a.
Infection/Sepsis, *n* (%)	4 (7.3%)	1 (3.3%)	3 (12.0%)	8.7%	−5.6–23.0%	0.218
Malignancy, *n* (%)	0 (0.0%)	0 (0.0%)	0 (0.0%)	0.0%	n. a.	n. a.
Thromboembolic event/bleeding, *n* (%)	0 (0.0%)	0 (0.0%)	0 (0.0%)	0.0%	n. a.	n. a.
All causes, *n* (%)	8 (14.5%)	1 (3.3%)	7 (28.0%)	24.7%	6.0–43.4%	0.010 *

AFL = atrial flutter; CI = confidence interval; HTX = heart transplantation; *n* = number; n. a. = not applicable; * = statistically significant (*p* < 0.050).

## Data Availability

The original contributions presented in this study are included in the article; further inquiries can be directed to the corresponding author.
